# Distensibility and Strength of the Pelvic Floor Muscles of Women in the Third Trimester of Pregnancy

**DOI:** 10.1155/2014/437867

**Published:** 2014-04-28

**Authors:** Carla Dellabarba Petricelli, Ana Paula Magalhães Resende, Julio Elito Júnior, Edward Araujo Júnior, Sandra Maria Alexandre, Miriam Raquel Diniz Zanetti, Mary Uchiyama Nakamura

**Affiliations:** ^1^Department of Obstetrics, Federal University of São Paulo (UNIFESP), Rua Carlos Weber, 956 Apartment, 113 Visage, 05303-000 Vila Leopoldina, SP, Brazil; ^2^Faculty of Physical Education and Physiotherapy, Federal University of Uberlândia (UFU), Uberlândia, MG, Brazil

## Abstract

*Objective.* The objective of this study was to compare the role of the pelvic floor muscles between nulliparous and multiparous women in the third trimester of pregnancy, by analyzing the relationship between electrical activity (surface electromyography—EMG), vaginal palpation (modified Oxford scale), and perineal distensibility (Epi-no). *Methods.* This was an observational cross-sectional study on a sample of 60 healthy pregnant women with no cervical dilation, single fetus, gestational age between 35 and 40 weeks, and maternal age ranging from 15 to 40 years. The methods used were bidigital palpation (modified Oxford scale, graded 0–5), surface EMG (electrical activity during maximal voluntary contraction), and perineal distensibility (Epi-no device). The Pearson correlation coefficient (*r*) was used to analyze the Epi-no values and the surface EMG findings. The Kruskal-Wallis test was used to compare the median values from surface EMG and Epi-no, using the modified Oxford scale scores. *Results.* Among the 60 patients included in this study, 30 were nulliparous and 30 multiparous. The average maternal age and gestational age were 26.06 (±5.58) and 36.56 (±1.23), respectively. It was observed that nulliparous women had both higher perineal muscle strength (2.53 ± 0.57 versus 2.06 ± 0.64; *P* = 0.005) and higher electrical activity (45.35 ± 12.24 **μ**V versus 35.79 ± 11.66 **μ**V; *P* = 0.003), while among the multiparous women, distensibility was higher (19.39 ± 1.92 versus 18.05 ± 2.14; *P* = 0.013). We observed that there was no correlation between perineal distensibility and electrical activity during maximal voluntary contraction (*r* = − 0.193; *P* = 0.140). However, we found a positive relationship between vaginal palpation and surface electromyography (*P* = 0.008), but none between Epi-no values (*P* = 0.785). *Conclusion.* The electrical activity and muscle strength of the pelvic floor muscles of the multiparous women were damaged, in relation to the nulliparous women, while the perineal distensibility was lower in the latter group. There was a positive relationship between surface EMG and the modified Oxford scale.

## 1. Introduction

In physiotherapeutic practice, the function of the pelvic floor muscles is evaluated in pregnant women in order to ascertain the ability of the muscles to perform muscle contraction and to identify possible dysfunctions [1]. With uterine growth, this region becomes overloaded and, through hormonal influence and biomechanical alterations of the pelvis, both muscle tone and muscle strength may decrease. This may result in urinary incontinence.

The main methods that have been described for evaluating the pelvic muscles are surface electromyography (EMG), which is the gold standard for studying electrical activity and has been validated in pregnant woman [2], and the bidigital palpation method (modified Oxford scale), which is simple, well-tolerated, minimally invasive, and widely used by physiotherapists.

During pregnancy, however, considering that there is a need for greater distensibility of the pelvic floor muscles during delivery, this question has been assessed using the Epi-no device. This device was developed by the company TECSANA in 1999 with the intention of being a vaginal dilator.

Some authors have reported that daily use of Epi-no from the 34th week of gestation to term reduces the incidence of episiotomy in primiparous women [3, 4], increases the incidence of an intact perineum [5], reduces the incidence of avulsion of the levator ani muscle, and reduces the incidence of microtrauma [6]. Zanetti [7] used this device to measure perineal distensibility during the first stage of delivery labor. After removing the inflated Epi-no from the vaginal canal, a measuring tape was positioned around the part with the largest diameter and the maximum circumference in centimeters was measured to ascertain how much stretching the muscle had acquired. In her study, there was a positive correlation with perineal integrity at the expulsive stage of delivery when the circumference was greater than 21 cm; that is, there would be no need for episiotomy because of the reduced risk of perineal laceration.

At the end of the pregnancy, it is expected that the pregnant woman will have pelvic floor muscles distensible enough for the passage of the fetus without the need for dilating surgery (episiotomy or perineotomy) and also that these muscles will not lose tone and strength, so that their function can be completely restored in the postpartum period. Therefore, the aim of this study was to compare the role of the pelvic floor muscles between nulliparous and multiparous women in the third trimester of pregnancy by analyzing the relationship between electrical activity (surface EMG), vaginal palpation (modified Oxford scale), and perineal distensibility (Epi-no).

## 2. Materials and Methods

This was an observational cross-sectional study with consecutive sampling that was carried out at the Prenatal Clinic of the Department of Physiological and Experimental Obstetrics, Federal University of São Paulo, Paulista School of Medicine (UNIFESP/EPM), from January 2012 to January 2013. The study was approved by the institution's Research Ethics Committee under number 513/10.

The study included 60 healthy pregnant women, aged 20–40 years (30 nulliparous and 30 multiparous), between their 35th and 40th weeks of pregnancy with a single fetus, with no cervical dilation, after they signed an informed consent statement. Patients who underwent an elective cesarean section in the current pregnancy or had a neurological deficit of the pelvic girdle, absence of perineal contraction, genital bleeding of any origin, or genitourinary tract infection were excluded from the study.

The women who agreed to participate in the study completed a questionnaire that asked for demographic and obstetric information. Following this, they were examined in the lithotomy position, with inspection of the pelvic floor muscles to test the ischiocavernosus, anal cutaneous, and cough reflexes, as well as to assess and guide them regarding contraction of the perineal muscles without the use of the abdominal, adductor, and gluteal muscles.

Surface electromyography of the pelvic floor muscles was performed using eight-channel surface electromyography equipment made by EMG System of Brazil (model 830C). This is a signal processer with bandpass filters, cutoff frequencies of 20–500 Hz, instrumentation preamplifier (20-fold gain), differential amplifier with bipolar input, and common mode rejection > 100 dB.

This device was coupled to an HP Pavilion DM4-2055br computer. The data were processed using specific software (EMGLab version V1.1) for signal acquisition in microvolts (*μ*V) and subsequent root mean square (RMS) analysis. A 12-bit A/D board was used to convert from analogue to digital signals at a sampling frequency of 2000 Hz.

**Figure 1:**
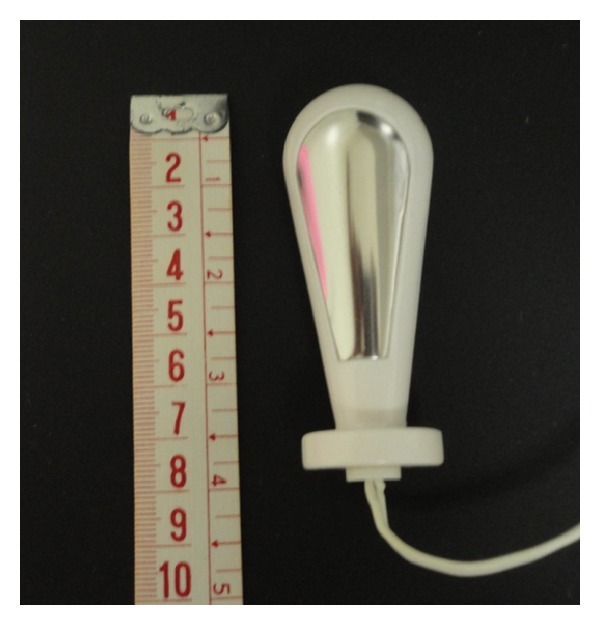
Vaginal probe used for recording myoelectric activity.

The electrical activity of the pelvic floor muscles was recorded using a cone-shaped intravaginal sensor (Chattanooga Group) with two opposing metal parts positioned on the side walls of the vagina (Figure 1). The silver chloride reference electrode (Medtrace 133) was placed on the lateral malleolus.

**Figure 2:**
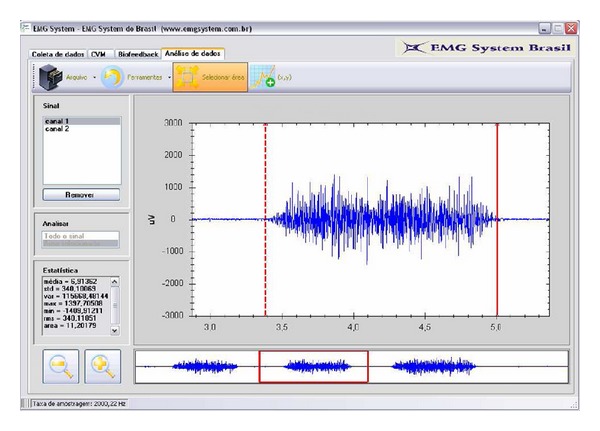
EMGLab1 software: exemplifying the selection of the best of three maximum voluntary contractions during the electromyography evaluation.

For the EMG recordings, the patient performed three maximum voluntary contractions, followed by relaxation, with 10 seconds of resting between the contractions. The best of the three contractions was selected for the study [2] (Figure 2).

Next, a clinical examination of muscle contractility was performed by means of bidigital vaginal palpation to a maximum depth of three inches. The muscle contractions were graded from 0 to 5 using the modified Oxford scale [8].

The perineal distensibility was then evaluated using the Epi-no device. After the deflated balloon had been inserted into a Microtex condom, lubricant gel was applied and it was introduced into the vagina such that only two centimeters of the distal portion of the device was outside of the introitus. The balloon was then gradually inflated until the limit tolerated by the patient was reached. The device was then slowly withdrawn, the condom was discarded, and the greatest circumference of the balloon was measured while it was still inflated.

For statistical analysis, the Minitab 16.1 software (State College, PA, USA) was used. Student's *t*-test was applied in relation to the variables of maternal age, gestational age, body mass index (BMI), surface EMG, and Epi-no; and the Mann-Whitney test was used for the number of pregnancies, the number of abortions, and the modified Oxford scale. The Pearson correlation coefficient (*r*) was used to analyze the Epi-no values and the surface EMG findings. The Kruskal-Wallis test was used to compare the median values from surface EMG and Epi-no using the modified Oxford scale scores. The significance level was set at *P* < 0.05.

## 3. Results

Out of the 60 patients included in this study, 30 were nulliparous and 30 were multiparous. The data relating to maternal age, gestational age, body mass index, number of pregnancies, and number of abortions are described in Table 1. It was observed that the multiparous women were older (*P* = 0.007) and had greater weight (*P* < 0.001).

In the multiparous group, 21 were primiparous (10 gave birth vaginally and 11 by cesarean section). The rest were secundiparous, of whom seven gave birth vaginally and two had both delivery methods.

We found that the two groups were similar regarding pelvic floor dysfunction, which was analyzed in this study as urinary incontinence complaints (63.33 versus 50.0%; *P* = 0.297).

Regarding the modified Oxford scale, surface EMG, and Epi-no evaluation methods, it was observed that, among the nulliparous women, both muscle strength and electrical activity were greater, while perineal distensibility was greater among the multiparous women (Table 2).

The Pearson correlation coefficient (*r*) showed that there was no relationship between the surface EMG findings and the Epi-no values (*r* = −0.193; *P* = 0.140).

**Figure 3:**
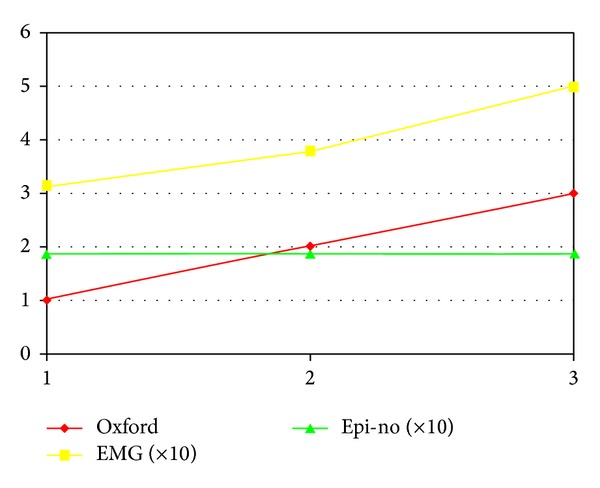
Correlation of the evaluation methods: positive between surface EMG and the modified Oxford scale and absence of relationship between the data from Epi-no and from the other methods.

Using the Kruskal-Wallis test, we found a positive relationship between the surface EMG findings and the modified Oxford scale scores. On the other hand, there was no correlation between the modified Oxford scale and perineal distensibility (Table 3 and Figure 3).

## 4. Discussion

Studying the pelvic floor during pregnancy allows for a greater understanding of this structure, so that it can be preserved during delivery, even if during pregnancy it undergoes alterations that result in urinary and fecal incontinence, along with sexual dysfunction.

It is important to emphasize that our patients were examined before undergoing any perineal distensibility test. If the electrical activity or muscle contractility was found to be lower during the study, it would show that the pregnancy itself was responsible for modifying muscle strength.

Based on recent published papers, we evaluated the role of the pelvic floor muscles in 60 pregnant women (30 nulliparous and 30 multiparous) by means of surface EMG [9, 10], bidigital palpation (modified Oxford scale) [11], and perineal distensibility (Epi-no) [7]. These methods are considered minimally invasive and can be safely used during pregnancy.

Because of the specific features of the equipment, surface EMG was the first method used in the evaluation in this study. In the maximum voluntary contraction evaluation, the nulliparous women presented higher scores for the electrical activity of the pelvic floor muscles than the multiparous women (45.35 ± 12.24 *μ*V versus 35.79 ± 11.66 *μ*V; *P* = 0.003). Our findings were similar to the findings from the study by Resende et al. [10], whose group of pregnant woman presented a result similar to that of our multiparous group (30.0 ± 19.4 *μ*V), and also to the findings of Botelho et al. [9], who analyzed 79 primiparous women in their third trimester of pregnancy and found an average of 35.4 *μ*V.

The difference between the groups may have been influenced by other factors that are considered to lead to a risk of pelvic floor muscle dysfunction, such as old age, number of pregnancies, parity, high body mass index (BMI), and presence of urinary incontinence in the third trimester of pregnancy. However, it is important to consider that pregnancy itself, excluding delivery labor, can reduce electrical activity, which modifies the function of the pelvic muscles, as was shown by Resende et al. [10], whose electromyographic recordings of maximum voluntary contractions from nulliparous women were much higher than those of the group of nulliparous women in the present study.

Bidigital palpation, graded using the modified Oxford scale, was the second evaluation method. This made it possible to observe the vaginal occlusion and pelvic floor muscle elevation and the patient's ability to perform correct contraction and muscle relaxation. Similar to the findings of surface EMG, we found that the nulliparous women also had greater muscle contractility than the multiparous women. In relation to the scale, scores of 2.0 refer to low-intensity contraction with little sustainment. According to the literature, patients who reach values of 3.0 onwards, without recruitment of other muscle groups, present proper muscle contraction [12].

Reduced muscle contractility may be associated with dysfunction of the pelvic floor muscles. Moen et al. [12] found that women with advanced age and high parity had lower scores during the evaluation, which agrees with our study.

To evaluate perineal distensibility, we used the Epi-no device, as shown in previous studies [5, 7]. At this stage, the multiparous women presented greater perineal distensibility than the nulliparous women (19.39 ± 1.92 versus 18.0 ± 2.14; *P* = 0.013). This procedure was performed around the 36th week of pregnancy, at which time the vaginal tissues and support tissues are mediated by hormones and, therefore, are more distended [13]. Other studies have shown that prolonged and difficult previous deliveries may result in increased degradation of the vaginal wall collagen, which leads to relaxation of this region [9, 14]. Shek et al. [15] reported the possibility that modifications that occurred in previous pregnancies would be permanent, especially regarding the hiatal dimension.

Similar to our study, Zanetti [7] found greater distensibility in the multiparous women than in the nulliparous women (20.7 ± 0.46 versus 19.3 ± 2.8 cm; *P* < 0.001) during the delivery labor. The two studies differ regarding the time at which they were conducted: in the present study, the pregnant women were evaluated two to four weeks before parturition, whereas, in Zanetti's study, data collection occurred over a period of effective delivery labor, which explains the difference of one centimeter of perineal distensibility.

Finally, we investigated the possible relationship between the assessment methods. Our results showed a positive correlation between the median values of surface EMG and the modified Oxford scale scores, such that the higher the recorded electrical activity was, the greater the muscle strength also was. Similar results were found in the studies by Botelho et al. [11] and Marques et al. [16].

Surface EMG does not evaluate muscle strength but, rather, it evaluates electrical activity promoted by recruitment of motor units. We were thus able to confirm what some authors have indicated regarding the good correlation between the number of activated motor units, increase in electromyography amplitude, and muscle strength [17–19].

On the other hand, we did not find any relationship between the assessment of perineal distensibility using Epi-no and the electromyography findings and modified Oxford scale scores. If perineal extensibility is taken to be an independent variable of both strength and myoelectric activity, it can be assumed that the fact that there is greater distensibility does not necessarily reduce muscle strength. We were able to affirm this over the range of circumferences from 13.5 to 24 cm, which were the minimum and maximum values obtained in our study. Further studies need to be conducted not only on this range of circumferences but also on strength and myoelectric activity, such as in some situations in which the tension decreases: for example, when the muscle fiber is kept at short lengths or beyond its resting length. In addition, situations of stretching of peripheral nerves (by more than 20% of their capacity) [20] or when there is muscle injury (laceration) [21] need to be further investigated.

Although there are no similar studies in the scientific literature, our findings corroborate the hypothesis that it is permissible to use Epi-no like a preparatory method for vaginal delivery, safely without damaging or impairing muscle strength (muscle contractility and/or electrical activity), at least up to the threshold circumference of 24 cm. Furthermore, through Epi-no, it is also possible to evaluate the real need for episiotomy and prevent its indiscriminate use, thereby protecting the patient from complications such as perineal pain in the immediate postoperative period and dyspareunia in the late puerperium. In addition, studies have shown that episiotomy does not protect women from the risk of urinary and fecal incontinence [22, 23].

Furthermore, we believe that the Epi-no can be used as a tool to assess the real necessity of episiotomy and prevent its indiscriminate use, protecting the patient from its complications. However, future studies in the postpartum period are necessary to prove our results.

## 5. Conclusion

The pelvic floor muscle function of multiparous women was lower than that of nulliparous women, regarding electrical activity and muscle strength. On the other hand, multiparous women showed greater distensibility. A positive relationship was identified between surface electromyography and the modified Oxford scale, but no significance was observed when perineal distensibility was assessed using Epi-no.

## Figures and Tables

**Table 1:** Patient distribution according to demographic characteristics.

Variable	Group	*n*	Mean	Standard deviation	Minimum	Maximum	Median	*P *
Age	Nulliparous	30	26.10	4.93	20.00	39.00	25.00	0.007*
	Multiparous	30	29.90	5.60	21.00	39.00	28.00	

Gestational age	Nulliparous	30	36.50	1.28	35.00	39.00	36.00	0.677*
	Multiparous	30	36.63	1.18	35.00	39.00	36.50	

BMI	Nulliparous	30	27.06	3.77	20.58	37.36	26.04	<0.001*
	Multiparous	30	31.79	5.33	23.31	44.53	30.52	

Number of pregnancies	Nulliparous	30	1.26	0.58	1.00	3.00	1.00	<0.001**
	Multiparous	30	2.60	0.93	2.00	5.00	2.00	

Number of abortions	Nulliparous	30	0.26	0.58	0.00	2.00	0.00	0.991**
	Multiparous	30	0.30	0.70	0.00	3.00	0.00	

BMI: body mass index.

*Student's *t-*test; **Mann-Whitney.

**Table 2:** Comparison of the values found using the modified Oxford scale, surface EMG during maximum voluntary contraction, and Epi-no.

Variable	Group	*n*	Mean	Standard deviation	Minimum	Maximum	Median	*P *
Oxford	Nulliparous	30	2.53	0.57	1.00	3.00	3.00	0.005*
	Multiparous	30	2.06	0.64	1.00	3.00	2.00	

sEMG MVC	Nulliparous	30	45.35	12.24	17.27	69.09	47.86	0.003**
	Multiparous	30	35.79	11.66	17.07	61.49	36.55	

Epi-no	Nulliparous	30	18.05	2.14	13.50	23.00	17.75	0.013**
	Multiparous	30	19.39	1.92	16.50	24.00	19.00	

sEMG MVC: surface electromyography during maximum voluntary contraction.

*Mann-Whitney; **Student's *t-*test.

**Table 3:** Comparison between the findings on the modified Oxford scale between surface EMG during maximum voluntary contraction and Epi-no.

Variable	Modified Oxford scale			Significance (*P*)
	1	2	3	
Median sEMG MVC (*n*)	31.31 (6)	37.09 (30)	49.98 (24)	0.008*
Median Epi-no (*n*)	18.50 (6)	18.75 (30)	18.75 (24)	0.785*

sEMG MVC: surface electromyography during maximum voluntary contraction.

*Kruskal-Wallis.
